# Effects of Toothpaste Containing 2% Zinc Citrate on Gingival Health and Three Related Bacteria—A Randomized Double‐Blind Study

**DOI:** 10.1002/cre2.70020

**Published:** 2024-11-04

**Authors:** Yujie Zhou, Yi Zhou, Binyou Liao, Xiaobin Chen, Yulong Niu, Biao Ren

**Affiliations:** ^1^ Hospital of Stomatology, Guangdong Provincial Key Laboratory of Stomatology Guanghua School of Stomatology, Sun Yat‐sen University Guangzhou China; ^2^ State Key Laboratory of Oral Diseases & National Clinical Research Center for Oral Diseases, West China Hospital of Stomatology Sichuan University Chengdu China; ^3^ Hawley & Hazel Chemical Co. (ZS) Ltd. Zhongshan China; ^4^ Key Laboratory of Bio‐Resources and Eco‐Environment of Ministry of Education, College of Life Sciences Sichuan University Chengdu China

**Keywords:** antibacterial, gingival health, zinc citrate

## Abstract

**Objectives:**

Gingivitis is the initial stage of periodontitis, one of the most common oral diseases and the primary cause of tooth loss. This study aims to evaluate the effect of toothpaste containing 2% zinc citrate on gingival health and the abundance of three bacteria related to gingivitis and periodontitis.

**Methods and Materials:**

Eleven volunteers with the same oral health status were randomly assigned to the treatment (*n* = 5) and control (*n* = 6) groups. The control group used fluoride toothpaste, while the treatment group used fluoride toothpaste supplemented with 2% zinc citrate for 3 months. The plaque index, gingival index, and bleeding index were measured at baseline (0 day), 3 weeks, and 3 months. Dental plaque from four areas of the mouth (FDI criteria) was collected at the same timepoints. A total of 132 dental plaque samples were analyzed using quantitative PCR (qPCR) to monitor the abundance of *Actinobacillus actinomycetemcomitans*, *Porphyromonas gingivalis*, and *Tannerella forsythia*.

**Results:**

Toothpaste containing 2% zinc citrate significantly lowered the gingival index and reduced gum bleeding but did not affect the plaque index. It also reduced the total abundance of the three bacteria related to gingivitis and periodontitis in dental plaque over a long‐term period.

**Conclusions:**

Toothpaste with 2% zinc citrate persistently improves gingival health and reduces the presence of gingivitis‐associated bacteria in dental plaque.

**Trial Registration:**

Chinese Clinical Trial Registry (Clinical trial registration no.: ChiCTR1900020592) (09/01/2019).

## 1 Introduction

Gingivitis is the initial stage of periodontitis which is one of the most common oral diseases and the main cause of tooth loss (Goel and Baral 2017; He and Farrell 2017; Kassebaum et al. 2014; Peres et al. 2019). There are many types of gingivitis, while the most common type is chronic gingivitis due to its high incidence, especially in the old population (Haas et al. 2019; Silva‐Junior et al. 2017). This type of gingivitis is caused by dental plaque on the tooth surface along the gingival margin. Gum bleeding and dental calculus usually result from this dental plaque (Chapple et al. 2015). The microecology of dental plaque is the major etiology of gingival or periodontal diseases (Colombo and Tanner 2019; Könönen, Gursoy, and Gursoy 2019).

Three bacteria in the dental plaque, *Actinobacillus actinomycetemcomitans* (*A. actinomycetemcomitans*, *Aa*), *Pophyromonas gingivalis* (*P. gingivalis*, *Pg*), and *Tannerella forsythia* (*T. forsythia*, *Tf*), are proved to be highly related to the gingival or periodontal diseases (Chen et al. 2019; Chigasaki et al. 2018; Jordan et al. 2016; Ng et al. 2016; Sun et al. 2017; Takahashi et al. 2019). *A. actinomycetemcomitans* can significantly increase the prevalence in treated progressive lesions than nontreated progressive lesions in active periodontitis (Slots et al. 1986). *P. gingivalis* is thought to be a keystone bacterium that can regulate the balance of oral microbiota to cause gingivitis or periodontitis (Olsen and Nichols 2018; Pritchard et al. 2017; Turnbaugh et al. 2007) and it can also produce virulence factors, such as fimbriae, gingipains, and lipopolysaccharides, to directly cause the inflammation of gingival or periodontal tissues (Filoche, Wong, and Sissons 2010). *T. forsythia* is often detected in the subgingival plaque of severe periodontitis (Monteiro et al. 2015). It can penetrate host cells or induce apoptosis by producing several virulence factors, such as a trypsin‐like protease and lipopolysaccharide (Settem et al. 2018). The detection rate of *Tf* from smokers is significantly increased (Joaquim et al. 2018). It has been recognized as one of the important gingival or periodontal pathogens (Demmer et al. 2015; Mombelli et al. 2017).

Modern oral health care products usually contain antiplaque agents in dentifrice formulations, which can significantly inhibit plaque formation and reduce gingival inflammation. Zinc salts, primarily zinc citrate, are widely used as antimicrobials in oral care products. Zinc ion can affect various oral microorganisms, resulting in the reduction of oral bacteria (ZnCl_2_), dental plaque (zinc acetate), dental caries (ZnCl_2_), and oral malodor (ZnCl_2_) (Giertsen and Scheie 1995; He, Pearce, and Sissons 2002; Watson, Cummins, and van der Ouderaa 1991). Saxton et al. suggested a dose‐dependent, plaque‐inhibiting effect of dentifrices containing both zinc citrate and triclosan. Reduction of plaque formation by toothpaste containing 0.5% zinc citrate and triclosan has been reported to range from 0% to 42% (Saxton, Lane, and van der Ouderaa 1987). Similarly, zinc citrate and triclosan containing dentifrice can effectively improve gingival problems such as plaque build‐up, gum bleeding, and swollen gums (Saxton and van der Ouderaa 1989; Schaeken et al. 1996; Stephen et al. 1990). The antibacterial mechanisms of zinc ions were dynamic. Zinc ions can accumulate on the bacterial cell wall to reduce the acidogenicity or decrease the nutrient transport into bacteria (Harrap, Saxton, and Best 1983). It can also change the potential of bacterial surfaces and then inhibit adhesion between bacteria and teeth (Gedalia et al. 1988).

Currently, most of the studies combine zinc ions and triclosan together to evaluate their effects on dental plaques. The triclosan is a strong antibacterial agent but the effects of zinc citrate itself on the gingivitis or periodontitis and the related pathogenic bacteria in the dental plaque, especially in a long‐term period, are still unclear. In this study, we aimed to investigate the effect of 2% zinc citrate‐containing toothpaste on gingival health and related pathogenic bacteria. Here, we accessed the plaque index (PLI), gingival index (GI), and bleeding index (BI) of the volunteers. Then, three gingivitis‐ or periodontitis‐related bacteria from dental plaques were monitored by quantitative PCR (qPCR) at different timepoint to investigate the long‐term effects of zinc citrate on oral bacteria.

## 2 Materials and Methods

### 2.1 Registration and Ethical Aspects

The study protocol was reviewed and approved by the Institutional Review Board (WCHSIRB‐D‐2014‐080) of West China Hospital of Stomatology and registered in the Chinese Clinical Trial Registry (Clinical trial registration no.: ChiCTR1900020592). This study used a simple randomization grouping method. This method was very easy to operate in small sample experiments and was suitable for situations where the number of research cases and the overall variability were both minimal. A total of 11 adult volunteers were recruited from West China Hospital of Stomatology (Table 1). Eleven volunteers (numbered 1–11) were randomly assigned to the treatment group and the control group using a method of simple random sampling. Examiners first assigned a unique number to each volunteer (1–11). Then, a random number generator was used to generate 6 unique random numbers between 1 and 11. The volunteers corresponding to these random numbers will form the control group, while the remaining volunteers will form the treatment group. Written informed consent was obtained from all participants in the study. The volunteers were enrolled in the study based on the inclusion and exclusion criteria. The examiners are specialists in periodontology at West China Hospital of Stomatology. They are all trained and calibrated. We confirm that this research was conducted in full accordance with the World Medical Association Declaration of Helsinki.

**Table 1:** Age and gender of the subject population.

Treatment	Number of subjects			Age	
	Male	Female	Sum	Mean	Range
Treatment	0	5	5	29.4	26–34
Control	2	4	6	27.83	25–31

*Note:* The following index results correspond to the sampling rules of plaque samples, and are averaged with the index results of each test quadrant.

### 2.2 Participants

Healthy adult male and female subjects were enrolled in the study based on the following inclusion criteria: (1) Subjects had to possess at least 20 uncrowned permanent natural teeth, in good general health without significant systemic diseases. (2) Subjects had to be between 18 and 65 years of age. (3) Pregnant or breastfeeding women were excluded from the study. (4) Subjects were required to present at baseline with PLI (Quigley and Hein) ≥ 2, GI (Loe–Silness) ≥ 1, BI (Mazza)≥ 1, Periodontal pocket depth ≤ 5 mm. (5) Individuals who participated in any other similar clinical study or panel test were excluded from the study. (6) Subjects who signed an informed consent form and completed clinical trials as required. The exclusion criteria were also employed: (1) Subjects who had allergic histories to ingredients of the test toothpaste. (2) With severe tartar. (3) Subjects who were currently taking drugs that could affect the test results. (4) Subjects who had taken antibiotics within 1 month before participating in the study. (5) Subjects who had open caries or lesions in the mucous membranes inside the oral cavity. (6) Subjects suffering from severe periodontitis. In this study, 11 participants completed all the study visits and tests.

### 2.3 Clinical Test Index

#### 2.3.1 Plaque

The plaque was scored according to the Turesky modification of the Quigley–Hein PLI, to record plaque coverage area, and the focus was to observe plaques near the gingival margin and interproximal plaques (Turesky, Gilmore, and Glickman 1970). PLI is the indicator used clinically to evaluate the Oral hygiene status and the effect after periodontal disease control. The PLI was scored as follows: 0 = No plaque; 1 = Separate flecks of plaque at the cervical margin; 2 = A thin, continuous band of plaque (up to 1 mm) at the cervical margin; 3 = A band of plaque wider than 1 mm, but covering less than 1/3 of the side of the crown of the tooth; 4 = Plaque covering at least 1/3, but less than 2/3 of the side of the crown of the tooth; 5 = Plaque covering 2/3 or more of the side of the crown of the tooth.

#### 2.3.2 Gingivitis

Gingivitis was scored according to the Löe–Silness GI (Löe and Silness 1963). GI was measured before measuring PLI by staining. GI is the indicator used clinically to evaluate the severity of symptoms such as gingival bleeding and swelling. The GI was scored as follows: 0 = Absence of inflammation; 1 = Mild inflammation—a slight change in color and little change in texture; 2 = Moderate inflammation—moderate glazing, redness, edema, and hypertrophy; 3 = Severe inflammation—marked redness and hypertrophy. Tendency for spontaneous bleeding.

#### 2.3.3 BI

The BI was scored according to the Mazza BI standard (Mazza, Newman, and Sims 1981). BI is the indicator used clinically to evaluate the severities of bleeding symptoms such as gingivitis and periodontal disease. The scoring criteria were as follows: 0 = Normal appearing, healthy gingival; 1 = Color change related to inflammation, but no bleeding; 2 = Slight bleeding that remained at the point of bleeding; 3 = Bleeding extending from the point of sampling and flowing around the gingival margin; 4 = Profuse bleeding that overflowed the gingival margin; 5 = Tendency to spontaneous bleeding.

### 2.4 Clinical Experimental Procedures

#### 2.4.1 Initial Screening

Subjects signed the *Clinical Trial Agreement* and agreed to participate in this study. The experiment recorded the subject's personal medical history and concomitant medication, followed by an oral soft tissue (OST) examination of the subject. In addition, the test evaluated the subject's erosion/abrasion/retraction (EAR), gum condition, loose teeth, and so forth. Subjects with frequent bleeding in daily life and moderate/severe gingivitis were included in the trial. The dentist evaluated the GI, BI, and PLI for subjects who were qualified for the basic oral condition. Subjects with a qualified index evaluation brushed their teeth with ordinary fluoride‐containing toothpaste and a soft‐bristled toothbrush under the guidance of the study site. The research organizers arranged for these subjects to receive dental cleaning and polishing and continued to brush their teeth with ordinary fluoride‐containing toothpaste at home for 3 weeks. In this study, the participants were randomly assigned to groups. A computer‐generated random number table was used to ensure that each participant had an equal chance of being assigned to different treatment groups, thereby minimizing selection bias. The study also employed a double‐blind design, meaning that both the participants and the researchers were unaware of the treatment assignments throughout the study.

#### 2.4.2 Collection of Experimental Samples

Three weeks later, the subjects received an evaluation of three indices (baseline) in the laboratory, BI and GI were examined first, followed by plaque staining, and PLI examination. Finally, the plaque was obtained. The teeth for plaque sampling were the periodontal index teeth (17, 16, 11, 26, 27, 37, 36, 31, 46, 47), and if the teeth in the periodontal index tooth were missing, the tooth with high inflammatory index in the quadrant was selected. Plaques were sampled at the site of inflammation: lingual and buccal (near‐middle, middle, and distal). A periodontal probe was used to scrape off all plaque on the inguinal hernia (squeeze clean as much as possible) with the assistant (using a sterile toothpick) to transfer the plaque to the vial. The sample tubes were kept clean and sterile before sampling and weighed. After sampling, the tubes were weighed to calculate plaque weight. The sample was stored in a refrigerator at −20°C and to be tested. Subjects were given a designated toothpaste after sampling. Subjects used a designated toothpaste to brush their teeth for 3 weeks and 3 months before going to the laboratory for index evaluation and extracting plaque and dental plaque photo records. Every visit required the inspection of soft and hard tissues. The treatment group was given Darlie Expert Gum Care toothpaste 1 (containing 2% zinc citrate and 1000 mg/kg F^−^), while the control group was given fluoride toothpaste without active ingredients (containing 1000 mg/kg F^−^). To reduce bias and enhance the reliability and validity of the research results, the packaging and appearance of the treatment and control groups were identical and were distributed and coded by an independent third party to maintain blinding during treatment and sampling.

### 2.5 Relative Quantification of Different Bacteria

The time‐dependent changes of three target bacteria (*Aa, Pg, Tf*) in the dental plaques from the treatment and control groups were examined by qPCR. The samples were combined from each quadrant into one sample. Total bacterial DNA were extracted from all samples with the following procedures: (1) lysozyme at a concentration of 1 mg/mL treated the samples at 37°C for 30 min; (2) the samples were treated with proteinase K at a concentration of 500 μg/mL for 30 min, then treated with 10% concentration of SDS at 37°C for 2 h; (3) CTAB (concentration of 1%, dissolved in 0.7 M NaCl) was added and the samples were treated in a 65°C water bath for 20 min; (4) phenol/chloroform/isoamyl alcohol (25:24:1) were added and shaken. It was centrifuged at 12,000 rpm for 15 min; (5) the supernatant was taken, chloroform/isoamyl alcohol (24:1) were added to the vortex, and centrifuged for 15 min at 12,000 rpm; (6) step 5 was repeated once again; (7) 1/10 volume 3 M sodium acetate and an equal volume of isopropanol was added and precipitated at −20°C for 30 min; (8) it was centrifuged for 15 min at 12,000 rpm, the supernatant was removed and washed twice with 70% ethanol. Ethanol was removed, after being dried in the oven, and re‐dissolved in 30–50 μL water; (9) low melting point agarose (LMP agarose) was cut and purified (optional).

Then, the qPCR was performed on a Roche 480 Real‐Time PCR and according to the TaKaRa's SYBR® Premix Ex TaqTM (TaKaRa Code: DRR041A) kit instructions. A two‐step PCR reaction procedure was used. The specific procedure was as follows: (1) 95°C for 30 s; (2): 40 PCR cycles (95°C 5 s, 60°C 30 s); different extension temperatures and times were determined based on different primers. Triplicate at each timepoint. All detection primer sequences are listed in Supporting Information S1: Table S1.

### 2.6 Statistical Processing

All data were assessed for normality using the Shapiro–Wilk test. The test results show that the data in each group meet the criteria for normal distribution (*p* > 0.05). The changes in PLI, GI, and BI were described by the mean values. The abundance of three bacteria was quantitated relative to the calibrator and was expressed as the median $-∆∆C_{t}$. The $-∆∆C_{t}$ values were plotted as violin plot and the variations between different groups were compared by one‐way analysis of variance (ANOVA). If the median of the group is bigger than 0 and *p* < 0.05, the bacterial abundance is significantly increased. On the contrary, if the median of the group is lower than 0 and *p* < 0.05, the abundance is significantly decreased. We conducted our data analysis using SPSS version 27.

## 3 Results

### 3.1 Zinc Citrate‐Containing Toothpaste Improved the Gingival Health

A total of 11 subjects were enrolled in the randomized double‐blind trial according to the inclusion and exclusion criteria (Table 1). No adverse reactions were found during the trial. At the baseline, there were no significant differences among the volunteers in PLI (Table 2), GI (Table 3), and BI (Table 4) when they started to enroll in this trial (*p* > 0.05).

**Table 2:** The effects of zinc citrate on the plaque index.

Group	Number of test quadrants	Baseline examination	Three‐week examination	Three‐month examination	∆ (Index reduction after 3 months compared to the baseline)
Treatment	20	2.55 ± 0.15	2.57 ± 0.17	2.52 ± 0.16	0.05
Control	24	2.58 ± 0.12	2.56 ± 0.17	2.53 ± 0.17	0.03

^a^Compared to the baseline, *p* < 0.05.

**Table 3:** The effects of zinc citrate on the gingival index.

Group	Number of test quadrants	Baseline examination	Three‐week examination	Three‐month examination	∆ (Index reduction after 3 months compared to the baseline)
Treatment	20	1.97 ± 0.10	1.68 ± 0.41^a^	1.87 ± 0.22^a^	0.10
Control	24	1.96 ± 0.11	1.82 ± 0.27^a^	1.94 ± 0.13	0.01

^a^ Compared to the baseline, *p* < 0.05.

**Table 4:** The effects of zinc citrate on the bleeding index.

Group	Number of test quadrants	Baseline examination	Three‐week examination	Three‐month examination	∆ (Index reduction after 3 months compared to the baseline)
Treatment	20	2.64 ± 0.37	2.12 ± 0.44^a^	2.25 ± 0.44^a^	0.39^b^
Control	24	2.55 ± 0.33	2.19 ± 0.33^a^	2.39 ± 0.44^a^	0.15

^a^ Compared with the baseline, *p* < 0.05.

^b^ Compared with the control, *p* < 0.05.

The toothpaste with or without zinc citrate had no significant effects on the PLI both at 3 weeks and 3 months (Table 2), indicating that zinc citrate has no effects on the biomass of dental plaque. The PLI reduction from the treatment group between 3 months and baseline was slightly larger than that from the control group, but there was no significant difference (Table 2).

After using the toothpaste with or without zinc citrate for 3 weeks, both treatment and control groups significantly reduced the GI (*p* < 0.05) (Table 3). Specifically, the GI reduced from 1.97 ± 0.10 to 1.68 ± 0.41 in treatment group (*p* < 0.05), while the GI reduced from 1.96 ± 0.11 to 1.82 ± 0.27 in the control group (*p* < 0.05) (Table 3). After 3 months, zinc citrate‐containing toothpaste can still improve the GI (1.87 ± 0.22, *p* < 0.05) (Table 3); however, the GI from the control group at 3 months was similar to the baseline (Table 3), indicating the long‐term effects of zinc citrate in gingival health. Although there was no significance between the treatment and control groups in the GI at 3 weeks and 3 months, the GI reduction from the treatment group was larger than that from the control group (Table 3).

For the BI, both toothpastes with and without zinc citrate can significantly reduce the bleeding at 3 weeks and 3 months compared to the baseline (*p* < 0.05) (Table 4). Specifically, the BI reduced from 2.64 ± 0.37 to 2.12 ± 0.44 (3 weeks) and 2.24 ± 0.44 (3 months) in the treatment group, while the BI reduced from 2.55 ± 0.33 to 1.82 ± 0.27 (3 weeks) and 2.39 ± 0.44 (3 months) in the control group (Table 4). Meanwhile, when compared to the control group, the treatment group significantly decreased the BI at 3 months (*p* < 0.05), and the BI reduction between 3 months and baseline from the treatment group was significantly larger than that from the control group (*p* < 0.05) (Table 4), indicating the long‐term efficacy of zinc citrate on gingival health.

### 3.2 Zinc Citrate Reduced Gingivitis‐ and Periodontitis‐Related Bacteria in the Dental Plaque

The unchanged PLI and improved gingival health between the treatment and control groups led to a hypothesis that zinc citrate may affect the gingivitis or periodontitis bacteria in the dental plaque. Therefore, we monitored the dynamic changes of *A. actinomycetemcomitans* (*Aa*), *P. gingivalis* (*Pg*), and *T. forsythia* (*Tf*) in the dental plaques collected at different timepoints. The gingival conditions of four areas of the mouth (FDI criteria) were checked (*n* = 44) and the dental plaques were collected from these areas (*n* = 132) at different timepoint. The abundance of the three bacteria showed no significant difference among different locations (FDI criteria) in the oral cavity (Supporting Information S1: Figure S1).

**Figure 1:**
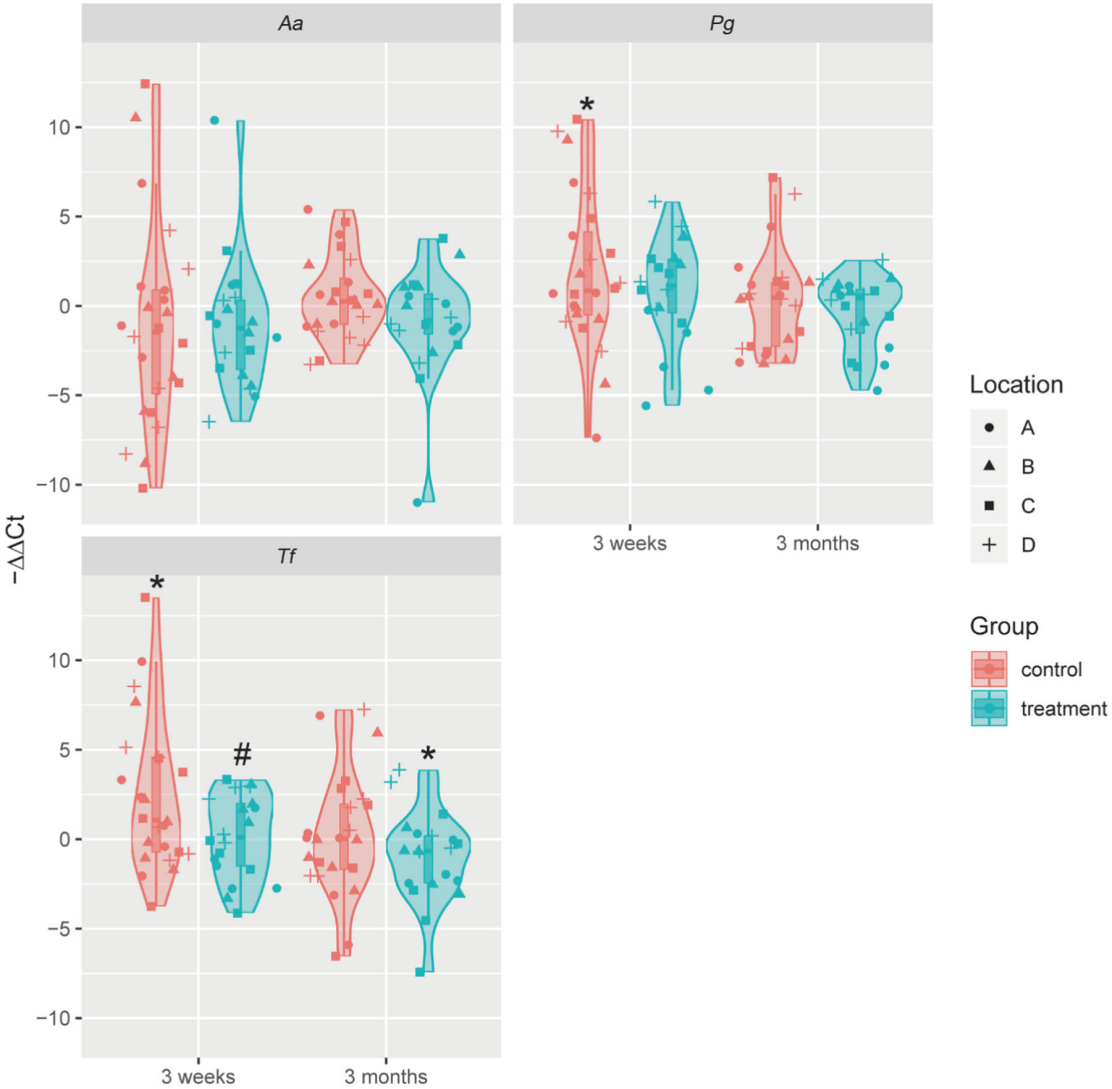
The abundance of the three bacteria in the dental plaques from the control and treatment groups at different timepoints. A, B, C, and D represent the four quadrants of the dental arch according to the FDI criteria. *p < 0.05 (3 weeks/months vs. baseline); ^#^ *p* < 0.05 (treatment vs. control).

When compared to the baseline, in the control group, *Tf* and *Pg* were significantly increased after 3 weeks, while *Aa* showed no difference (Figure 1). All three bacteria recovered to similar levels to that at the baseline after 3 months (Figure 1). In the treatment group, the abundance of three bacteria *Tf*, *Pg*, and *Aa* remained similar to the baseline after 3 weeks (Figure 1). Meanwhile, the abundance of *Tf* was significantly decreased after 3 months in the treatment group (Figure 1). When compared with the control group, the treatment group significantly reduced *Tf* at 3 weeks (Figure 1). These results indicated that zinc citrate‐containing toothpaste can reduce the overgrowth of pathogenic bacteria in the dental plaque for a long‐term period without the effect on the biomass.

**Figure 2:**
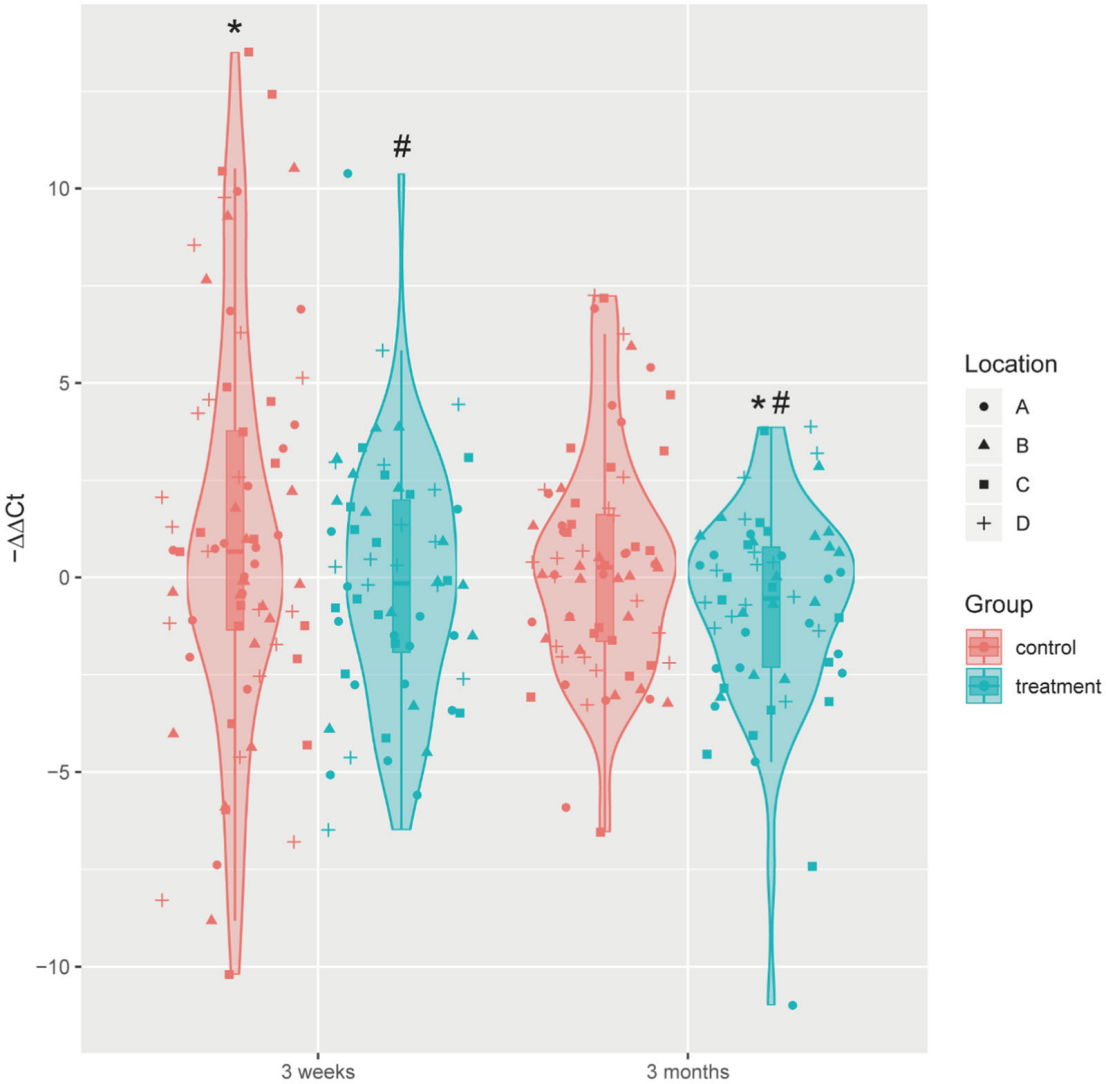
The abundance of the “core bacteria” in the dental plaques from the control and treatment groups at different timepoints. A, B, C, and D represent the four quadrants of the dental arch according to FDI criteria. * *p* < 0.05 (3 weeks/months vs. baseline); ^#^ *p* < 0.05 (treatment vs. control).

Then, we combined all three bacteria as “core bacteria” to analyze the effects of zinc citrate. In the control group, the “core bacteria” significantly increased after 3 weeks and became similar to the baseline after 3 months (Figure 2). In the treatment group, the abundance of the “core bacteria” was similar to the baseline after 3 weeks but significantly reduced after 3 months (Figure 2), indicating the long‐term effect of zinc citrate on the structure of the dental plaque. When compared with the control group, the treatment group significantly decreased the “core bacteria” after both 3 weeks and 3 months (Figure 2), in line with the long‐term effect of zinc citrate on gingival health (Tables 3 and 4).

## 4 Discussion

Zinc citrate is a common ingredient in oral care products, known for its significant antibacterial properties (Uwitonze et al. 2020). Its antibacterial mechanism includes releasing zinc ions that disrupt the structure of bacterial cell membranes, inhibiting the normal function of bacterial enzymes, preventing bacterial adhesion, and inhibiting biofilm formation. Through its direct bactericidal effects and indirect actions by preventing bacterial adhesion and biofilm formation, zinc citrate serves as an effective antibacterial component in oral care products (Gudkov et al. 2021; Zhang et al. 2021). In practical applications, the optimal concentration of zinc citrate should be determined based on the product's formulation, intended use, and regulatory requirements. The 2% concentration selected for this experiment is based on the sensitivity of oral bacteria to zinc ions, the effectiveness and safety of its application in the oral cavity, and the optimal ratio with other components in the toothpaste. A 2% concentration can provide sufficient zinc ions to inhibit the growth of oral bacteria while not causing irritation or toxicity to oral tissues.

The PLI, GI, and BI are classical and applicational clinical indexes to determine gingival health (Armitage 2004; Fine 1992; Mombelli 2003; Reddy 1997; Wadia and Chapple 2019). Gingival inflammation can be judged by observing the color, shape, and texture of the gums and whether the bleeding is detected after the diagnosis. According to our results, both the control and treatment groups were able to reduce the GI after 3 weeks. However, the control group returned to baseline levels, while the treatment group can still reduce the GI after 3 months indicating the stability and long‐term effects of zinc citrate. Meanwhile, both the treatment and control groups reduced the bleeding even after 3 months, but the treatment group reduced more compared to the control group indicating the strong inhibition of gingival bleeding by zinc citrate. Plaque biofilm is the basis for the survival, metabolism, and pathogenesis of oral bacteria, and is the initiator of gingival or periodontal diseases. PLI mainly reflects the hygiene of the mouth. Interestingly, in our study, both of the treatment and control groups showed no changes compared to the baseline based on this index.

*Aa*, *Pg*, and *Tf* are closely related to clinical parameters of gingival or periodontal diseases, especially the periodontal pocket depth and probing bleeding (Mombelli 2018; Uraz et al. 2019). It is considered to be the “advancing front” of periodontitis (Caton et al. 2018; Kay, Kramer, and Visser 2019; Visser and Ellen 2011; Zijnge et al. 2010). The unchanged PLI indicated that the components of dental plaque may be changed, especially the disease‐related bacteria. As expected, the treatment group significantly reduced the three bacteria as a whole after both 3 weeks and 3 months when compared to the control group indicating the strong antibacterial effects of zinc citrate and it may selectively reduce some species, such as *Tf*, in the plaque (Borsanelli et al. 2018). The decrease of the three bacteria and the unchanged PLI in our study also suggested that the abundance of the target pathogens in dental plaque is more critical on the response for determination of gingival health but not only the amount of dental plaque (Kageyama et al. 2017).

In this study, to increase the internal validity and reduce the biases of our results due to the small sample size, we performed strict methodological improvements. First, we used a computer‐generated random number table to ensure that each participant had an equal chance of being assigned to different treatment groups, thereby minimizing selection bias. This method was very easy to operate in small sample experiments and was suitable for situations where the number of research cases and the overall variability were both minimal. Additionally, the study also employed a double‐blind design. Both the participants and researchers were unaware of the treatment assignments throughout the study. Specifically, the packaging and appearance of the treatment and control groups were identical and were distributed and coded by an independent third party to maintain blinding during treatment and sampling. This design aimed to reduce bias and enhanced the reliability and validity of the research results. Furthermore, the examiners are specialists in periodontology at West China Hospital of Stomatology. They are all trained and calibrated. The involvement of professionals helps to enhance the precision of the examination results and reduce experimental bias. Finally, the current statistical method for data analysis was ANOVA. We have conducted normality tests using the Shapiro–Wilk test to assess the normality of our data. These strict statistical methods increased the internal validity of our results.

Currently, although our results have provided some significant effects of zinc citrate on gingival health and key pathogens from the dental plaque, this study also had some limitations, specifically including the following aspects: (1) Sample size: a larger sample size could make our conclusions more solid as a small sample size might affect the reliability and generalizability of the results. Additionally, including more male participants could help eliminate the influence of gender on the experimental results. (2) Study duration: the longest duration in this study was 3 months. Whether zinc citrate could provide a long‐term effects more than 3 months needs further observations. (3) Environmental and dietary factors: in our current experiment, we did not consider factors such as different regional environments and dietary habits, and how these factors affect the activities of zinc citrate could not be conclude in this study. Our results have proved the primary but important effects of zinc citrate on gingival health and key pathogens in a small sample size, while more studies based on large sample size, long‐term measurement, different regional environments, dietary habits, and so on need to be further explored to confirm the clinical effects of 2% zinc citrate toothpaste on gingival health.

## Author Contributions

Yujie Zhou contributed to data acquisition, analysis, and interpretation and drafted the manuscript. Yi Zhou and Binyou Liao contributed to data acquisition, analysis, and interpretation and critically revised the manuscript. Biao Ren contributed to the conception and design and critically revised the manuscript. Xiaobin Chen contributed to data interpretation and critically revised the manuscript. Yulong Niu and Biao Ren contributed to the conception, design, data analysis, and interpretation, and drafted and critically revised the manuscript. All authors gave final approval and agreed to be accountable for all aspects of the work.

## Ethics Statement

The study was reviewed and approved by the Institutional Review Board (WCHSIRB‐D‐2014‐080) of West China Hospital of Stomatology and registered in the Chinese Clinical Trial Registry (Clinical trial registration no.: ChiCTR1900020592). All applicable international guidelines for the care and use of participants' samples were followed.

## Consent

All authors have reviewed the final version of the manuscript and have approved it for publication.

## Conflicts of Interest

The authors declare no conflicts of interest.

## Supporting information

Supporting information. **Table S1. Detection primers. Fig S1. The abundance of the three bacteria at different locations in oral cavity.** A, B, C, and D represent the four quadrants of the dental arch according to the FDI criteria

## Data Availability

All data generated or analyzed during this study are included in this published article (and its Supporting Information files).
