# Supplementary material for: Effects of Toothpaste Containing 2% Zinc Citrate on Gingival Health and Three Related Bacteria—A Randomized Double‐Blind Study
Source: Clin Exp Dent Res. 2024 Nov 4;10(6):e70020. doi: 10.1002/cre2.70020 (PMC11534642; doi:10.1002/cre2.70020)
Supplement: Supplementary file 1 — Supporting information. Table S1. Detection primers. Fig S1. The abundance of the three bacteria at different locations in oral cavity. A, B, C, and D represent the four quadrants of the dental arch according to the FDI criteria [file CRE2-10-e70020-s001.docx]

**
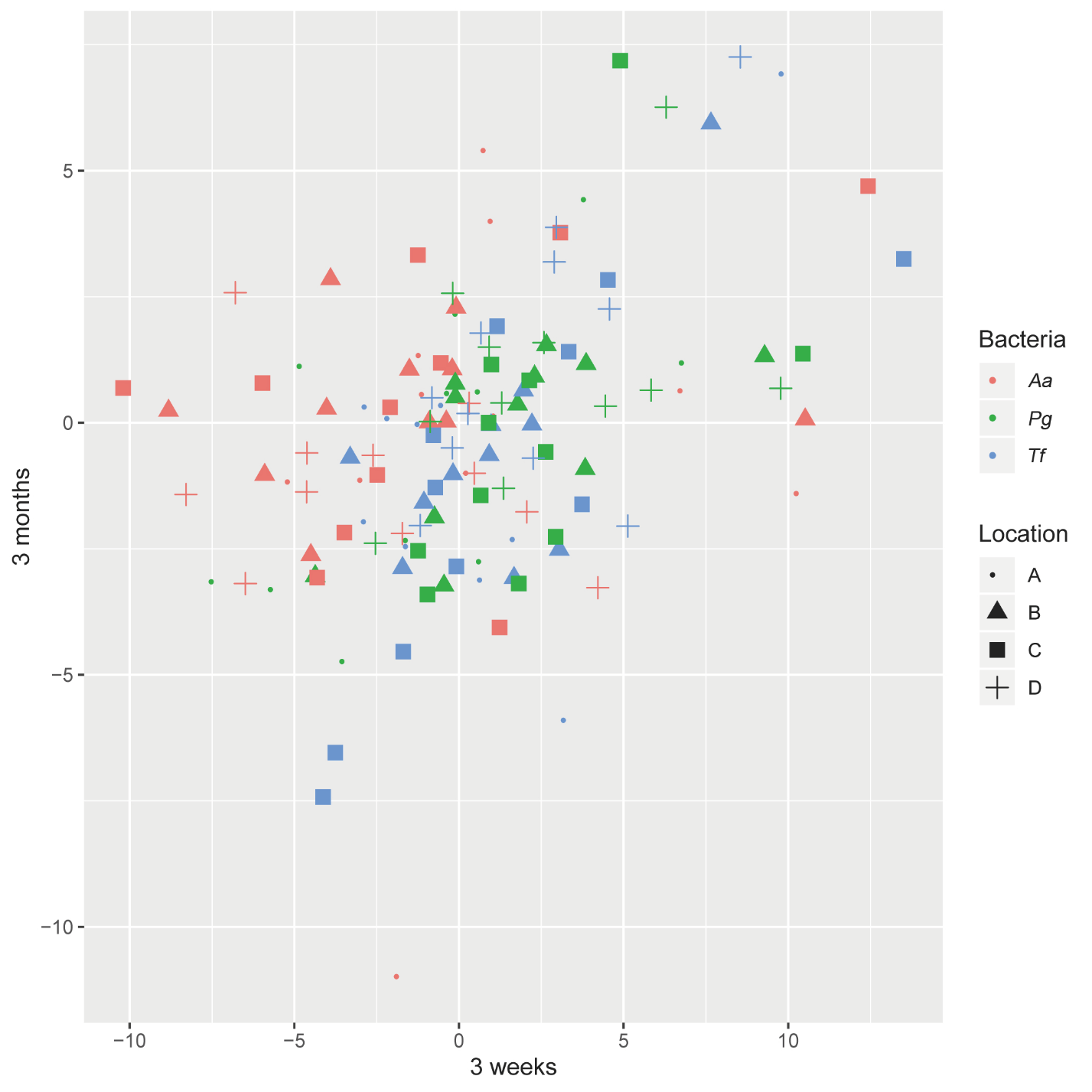
**

**Fig S1. The abundance of the three bacteria at different locations in oral cavity.**

A，B，C and D represent the four quadrants of the dental arch according to the FDI criteria

**Table S1. Detection primers**

| Strain | Specific primers | Amplicon (bp) |
| --- | --- | --- |
| *Aa* | AaF: ATTGGGGTTTAGCCCTGGTG | 360 |
|  | AaR: ACGTCATCCCCACCTTCCTC |  |
| *Pg* | PgF: TGTAGATGACTGATGGTGAAAACC | 197 |
|  | PgR: ACGTCATCCCCACCTTCCTC |  |
| *Tf* | TfF: AGCGATGGTAGCAATACCTGTC | 88 |
|  | TfR: TTCGCCGGGTTATCCCTC |  |
